# Assembly of respiratory syncytial virus matrix protein lattice and its coordination with fusion glycoprotein trimers

**DOI:** 10.1038/s41467-024-50162-x

**Published:** 2024-07-14

**Authors:** Bryan S. Sibert, Joseph Y. Kim, Jie E. Yang, Zunlong Ke, Christopher C. Stobart, Martin L. Moore, Elizabeth R. Wright

**Affiliations:** 1 Department of Biochemistry, University of Wisconsin, Madison, WI USA; 2 Cryo-Electron Microscopy Research Center, Department of Biochemistry, University of Wisconsin, Madison, WI USA; 3 Midwest Center for Cryo-Electron Tomography, Department of Biochemistry, University of Wisconsin, Madison, WI USA; 4 Department of Chemistry, University of Wisconsin, Madison, WI USA; 5 Department of Molecular Biosciences, University of Texas at Austin, Austin, TX USA; 6 Department of Biological Sciences, Butler University, Indianapolis, IN USA; 7 Meissa Vaccines, Inc., Redwood City, CA USA; 8 Morgridge Institute for Research, Madison, WI USA

**Keywords:** Cryoelectron tomography, Virus structures

## Abstract

Respiratory syncytial virus (RSV) is an enveloped, filamentous, negative-strand RNA virus that causes significant respiratory illness worldwide. RSV vaccines are available, however there is still significant need for research to support the development of vaccines and therapeutics against RSV and related *Mononegavirales* viruses. Individual virions vary in size, with an average diameter of ~130 nm and ranging from ~500 nm to over 10 µm in length. Though the general arrangement of structural proteins in virions is known, we use cryo-electron tomography and sub-tomogram averaging to determine the molecular organization of RSV structural proteins. We show that the peripheral membrane-associated RSV matrix (M) protein is arranged in a packed helical-like lattice of M-dimers. We report that RSV F glycoprotein is frequently observed as pairs of trimers oriented in an anti-parallel conformation to support potential interactions between trimers. Our sub-tomogram averages indicate the positioning of F-trimer pairs is correlated with the underlying M lattice. These results provide insight into RSV virion organization and may aid in the development of RSV vaccines and anti-viral targets.

## Abstract (web summary)

Here the authors use cryo-electron tomography and sub-tomogram averaging to determine the molecular organization of RSV. The structures show that the matrix (M) protein packs as a helical-like lattice and the F glycoprotein is present as anti-parallel pairs of trimers and coordinates with the M lattice.

## Introduction

Respiratory syncytial virus (RSV) is a negative-strand RNA virus in the order *Mononegavirales*, which includes many pathogenic viruses such as measles virus, rabies virus, and Ebola virus that can cause severe disease and death^1^. RSV causes respiratory illness in both children and adults and is estimated to cause over three million hospitalizations annually^2,3^. Two vaccines received FDA approval for use in older adults in 2023^4,5^. Prophylactic treatment with a monoclonal antibody targeting the RSV F protein is effective for preventing severe illness in high-risk children^6^. However, further research is needed to develop effective vaccines and therapeutics for all at risk populations.

RSV virions are enveloped, filamentous, and highly pleiomorphic in nature. Virions can range in length from less than one to over ten micrometers and virions also vary in diameter, with an average diameter of 130 nm^7^. Virion morphology and fitness may be altered by heat or mechanical stress during virus purification^8,9^. RSV has three surface proteins, fusion glycoprotein (F), attachment glycoprotein (G), and small hydrophobic protein (SH). RSV F is a class I fusion protein present as a homotrimer on the viral surface. The F protein has long been a promising target for vaccine development due to its sequence conservation between strains and the prevalence of neutralizing antibodies against prefusion-F^10–12^. This promise was validated by the release and FDA approval of two prefusion F based vaccines^4,5^. Structures for purified pre- and post-fusion F-trimers have been solved using x-ray crystallography and cryo-EM single particle analysis^11,13,14^. Several structures and conformational states of RSV F on intact virions have been determined, to a lower resolution, using cryo-electron tomography (cryo-ET) and sub-tomogram averaging (STA)^9,15^. RSV G is the primary attachment protein, although it has been shown to be non-essential for infection in some cell lines^16^. G can mediate attachment through heparin sulfate and the protein CX3CR1^17^. RSV SH is a small hydrophobic protein that has been shown to have viroporin activity^18^. SH is not required for viral entry or replication^19^.

RSV matrix (M) is a peripheral membrane-protein lining most of the interior of the inner viral membrane^7,15,20^. The presence of matrix, or a functionally similar protein, is widely conserved amongst negative-strand RNA viruses. Many studies of these viruses have identified a central role for matrix or matrix-like proteins in virion organization and assembly^21–24^. Correspondingly, RSV M is essential for assembly of filamentous virions and virus-like particles (VLPs)^25^. Purified RSV M has been crystallized as both a monomer and a dimer leaving some uncertainty regarding M organization in virions^26,27^. However, assembly of filamentous virions or virus-like particles (VLPs) is inhibited by mutations that disrupt M dimerization^26^. We, and others, have previously published results from cryo-ET studies and STA of RSV virions indicating that M is arranged in a lattice of dimers^20,28,29^. Assembly of a membrane-associated matrix or matrix-like protein lattice has been shown for several other viruses^30–36^. While RSV M alone is not sufficient for filamentous VLP assembly, only RSV M, F, and P (phosphoprotein) are required^37^.

Other RSV structural proteins include RSV P, N, M2-1, and L. RSV P is a non-catalytic phosphoprotein that is essential for viral RNA synthesis. It’s exact role in virion or VLP assembly remains unknown, but it is known to interact directly with M^38^. P interacts with other structural proteins as well, including L and M2-1, and may be involved in mediating interactions between M and other proteins^37–42^. Cryo-ET and super-resolution fluorescence microscopy demonstrated that M2-1 was present as regularly spaced densities between the M layer and the RNP in virions^8,15^. The role of M2-1 in virion structure remains uncertain, it has been suggested that RSV M associates with the nucleoprotein (N) through mutual neighboring interactions with M2-1^43^, though incorporation of N into VLPs does not require M2-1^26^. M2-1 has been shown to function as an anti-termination factor during RNA transcription and may have additional post-transcriptional functions as well^44–46^. RSV N is associated with the genomic RNA in a left-handed helical nucleocapsid^47,48^ that can be observed throughout the virion interior. The large RNA-dependent RNA polymerase (L) is associated with N in the viral filaments^49^.

To study the native structure of RSV and other enveloped viruses, we and others use whole-cell cryo-ET^20,30,50–52^. Cells are grown and infected with RSV directly on TEM grids^7,53,54^. The grids are then plunge-frozen to rapidly cryo-preserve the cells, associated viruses, and released virions in vitreous ice. By whole-cell cryo-ET, RSV virions are predominantly filamentous; however, irregular, branched, and bent viruses may still be observed^7,8^. Previous cryo-ET and STA studies have shown that RSV M is arranged in a helical-like lattice along the interior of the viral envelope^20,28,55^.

In this work we present high-resolution sub-tomogram averages of the RSV M lattice that clearly illustrate that the helical-like lattice is composed of an extended network of M-dimers. The 4.6 Å resolution sub-tomogram average of the M lattice allows for well correlated modeling of an M-dimer crystal structure into the averaged density. We further show that the position of F on the viral surface is ordered relative to the underlying M lattice. F is frequently observed in pairs (i.e., a dimer-of-trimers) on the viral surface and STA of F pairs indicates an anti-parallel arrangement of the F trimers with potential quaternary interactions between the two trimers of a bundle. A more complete understanding of RSV M and F interactions and organization is critical to inform future vaccine design and development.

## Results

### RSV F is arranged in rows and pairs on virions

**Fig. 1:**
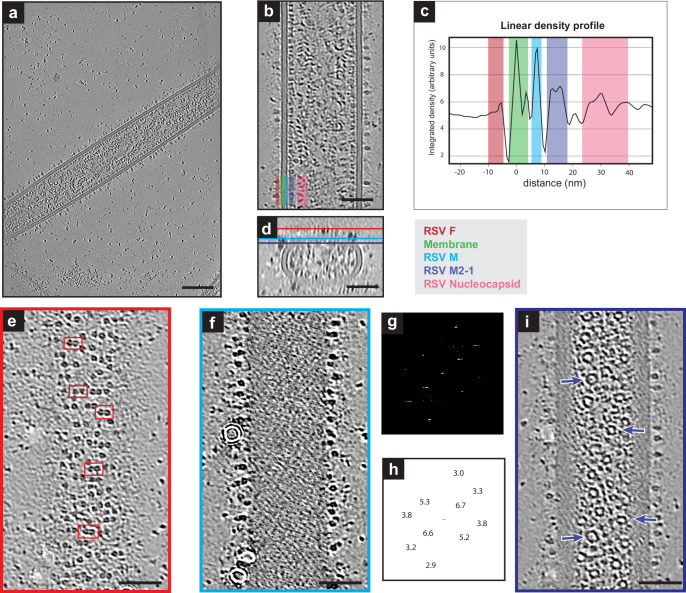
Organization of RSV structural proteins. **a** Single z-slice from a representative cryo-tomogram of RSV virions (*n* = 27). Protein density is black. **b** A reoriented Z-projection (5 nm thick) from a tomogram of an RSV virion (*n* = 33 virions, *n* = 27 tomograms). In this orientation, the viral membrane and several structural proteins are localized within columns in the image as indicated by the colored bars in the bottom left. Red, Green, Cyan, Purple, and Pink correspond to the positions of RSV F, membrane, RSV M, RSV M2-1, and nucleocapsid respectively as indicated to the under panel **c**. **c** An average linear density profile across the left-side of the virion in (**b**) showing the peaks corresponding with each column of proteins or membrane. Colored boxes correspond to the legend and positions indicated in **b**. Source data are provided as a Source Data file. **d** Y-projection (9 nm thick) of X, Z slices of the virion in **b**, Z-position of slices in (**e**–**g**) are indicated by the red, cyan, and purple bars respectively (*n* = 33). **e** Single slice from the virion in (**b**) showing RSV F on the top of the virion in the X, Y plane (red bar in (**d**) indicates position of slice in Z direction). Select examples of pairs of density are highlighted by red boxes (*n* = 33). **f** Single slice from the M protein layer from the virion in **b** (cyan bar in **d**) (*n* = 33). **g** FFT of the M layer from the same filament and z-slice shown in (**f**) from a weighted-back projection tomogram (no denoising) (*n* = 33). **h** The frequency (nm^−1^) of select peaks in the amplitude spectrum, determined by local maxima, is displayed in positions corresponding to the peaks in **g**. **i** Single slice from the virion in (**b**) showing the M2-1 layer (purple bar in **d**). Arrows point to ring like densities (*n* = 33). All slices from a binned by 4 tomogram (6.772 Å/px) denoised with cryoCARE. 500 nm scale bar for **a**, 50 nm scale bar for **b**, **d**, **e**, **f**, and **i**.

To determine the organization of RSV structural proteins on intact virions, we performed whole-cell cryo-ET of RSV virions released from infected cells that were grown directly on TEM grids. We used conditions like those previously outlined by Ke et al.^7^. Briefly, BEAS-2B cells were cultured directly on gold TEM grids and infected with RSV-A2mK+ at a multiplicity of infection (MOI) of 10. The cells and viruses on the grids were cryo-fixed by plunge-freezing 24 hours post infection. A slice from a representative tomographic volume is shown in Fig. 1a. Consistent with previous reports^7,53,56^, we were able to identify densities attributed to the viral membrane, the fusion (F) glycoprotein, matrix (M) protein, M2-1, and ribonucleoprotein (RNP) complex (Fig. 1b). RSV F was present as densities decorating the surface of the outer viral membrane. The M protein was closely associated with the membrane and appears as a continuous layer beneath the membrane (Fig. 1b). Interior to that was a layer of regularly spaced densities that has been attributed to M2-1^8^, though the presence of additional macromolecules in this layer cannot be ruled out. The RNP was frequently observed running along the M2-1 layer (Fig. 1b) as presented in the density profile plot (Fig. 1c). However, the RNP was not always closely associated with the M2-1 layer and was also be found throughout the interior of the virus, consistent with our previously published results^8^.

Upon closer examination of the distribution of RSV F along the surface of the virions, it was clear that F was not present as a tightly packed lattice, however, the distribution did not appear random. RSV F densities on the surface of virions appeared to be roughly organized into rows perpendicular to the long axis of the virus (Fig. 1e). Within these rows, F often occurred as pairs, i.e., a dimer-of-trimers, in which two trimers were closer to one-another than neighboring trimers (Fig. 1e, red boxes).

### Lattice density is present in M-layer of RSV virions

In tomograms of RSV where a continuous M layer persisted (Fig. 1b), we observed large regions of lattice-like density extending along the virions (Fig. 1f). The presence of this layer was more apparent when the virion was reoriented to be flat to the viewing plane allowing a larger surface of the M layer lattice to be viewed within a single section. The lattice-like nature within the layer was further confirmed by analyzing the amplitude spectrum from FFT analysis of the M protein layer (Fig. 1g). Peaks were present as short horizontal lines and local maxima analysis was used to identify the brightest positions for measurement. The peaks were found to correspond to frequencies of 6.7 nm^−1^, 5.3 nm^−1^, 3.8 nm^−1^, 3.3 nm^−1^, and 2.9 nm^−1^. Minor differences in the measured frequency of mirrored peaks were all significantly less than one pixel (0.676 nm, bin four) and most likely due to limited precision of the measurements. These peaks were later confirmed to be from the M lattice by validating that the same peaks were observed in the FFT analysis of a sub-tomogram average of the M-lattice (Supplementary Fig. 1).

Densities with a regular spacing of ~12.6 nm have been previously reported for M2-1^8^ and similar periodic densities were observed in virions at that layer (Fig. 1b, purple bar). Ring-like structures were observed in views from the top corresponding to the same distance from the membrane. There was no obvious organization to the rings. Some of the rings appeared to be consistent in size and shape with nucleocapsid^47^, however, they were not associated with longer strands of nucleocapsid. Additionally, variation in the ring diameter was noted, though this could be due, in part, to varied orientation of the rings relative to the tomographic slice.

### Sub-tomogram averaging of M-lattice

**Fig. 2:**
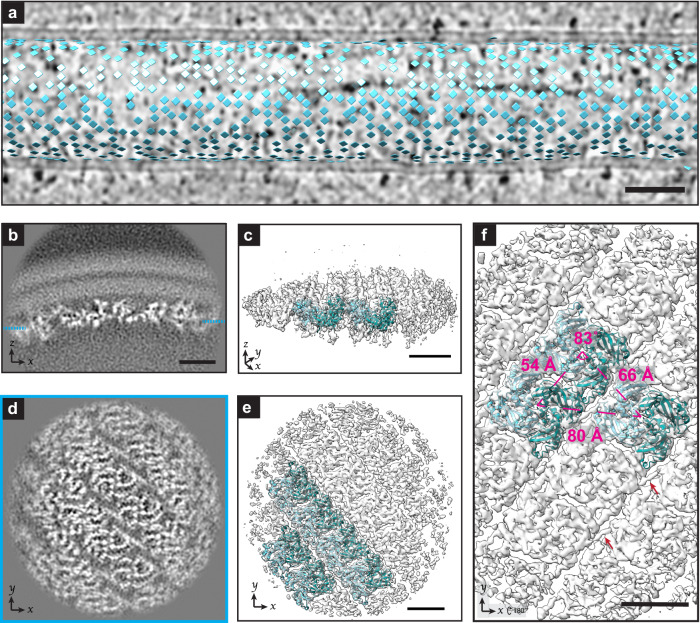
Sub-tomogram average of RSV M. **a** Model points for the center of sub-tomograms used in sub-tomogram averaging of RSV M are shown as squares and positioned above a tomographic slice from the corresponding virion segment (binned by 6 IsoNet processed volume) (*n* = 33). **b** Single slice from a sub-tomogram average of RSV M oriented with the M lattice shown underneath the viral membrane. Dashed lines indicate the z-position of the slice in **d**. **c** Isosurface rendering of RSV M sub-tomogram average with model fitting of the M dimer (PDB: 4v23). Models are shown as ribbon diagrams with the individual monomers differentially colored (dark blue and cyan). **d** Single slice from sub-tomogram average from **b**. **e** Isosurface of RSV M as viewed from the membrane toward the viral interior as in (**d**) with models of the M-dimer fit in to better visualize lattice organization. **f** Isosurface of M layer with model fitting of the M-dimer viewed from viral interior overlaid with the average center-to-center distances between individually fit dimers as well as the angles between dimers. Source data are provided as a Source Data file. Red arrows point to examples of map density between dimers that is not occupied by fitting 4v23. Density map in (**b**–**f**) is an average from *n* = 38,771 sub-tomograms. 50 nm scale bar for **a**, 5 nm scale bar for **b**–**f**.

We applied STA to analyze the organization of RSV M in the lattice (Fig. 2, Supplementary Movie 1). Figure 2a shows a tomographic slice of a virion segment overlaid with model points marking the centers of aligned sub-tomograms included in the average. The lattice organization was seen in the distribution of the points, although small gaps were present throughout. The gaps in model points did not definitively indicate gaps in the M lattice because some points may have been missed during particle selection. A tightly packed lattice associated with the inner leaflet of the viral membrane was seen in the sub-tomogram average density map (Fig. 2b, d). The organization and spacing of the lattice were particularly evident when oriented flat in the viewing plane (Fig. 2d). To validate that the lattice structure observed in the sub-tomogram average was consistent with a lattice of M-dimers, atomic models of the M dimer crystal structure (PDB: 4v23^26^) were fit into the average (Fig. 2c, e, f). Q-score analysis^57^ was done using the central four dimers to analyze the quality of fit. The average Q-residue score was 0.2737, less than the expected Q-score at 4.6 Å of 0.3319, however the Q-backbone score was 0.3681 (Supplementary Fig. 2). The M monomers within each dimer have been bicolored cyan and dark blue to better visualize the organization and packing. In addition to the densities in which the PDB: 4v23 model fits into, additional density connecting the dimers was seen on the interior side. Select areas of this density are indicated by red arrows in Fig. 2f and additional views are shown in Supplementary Fig. 3.

To measure the lattice spacing, UCSF Chimera^58^ was used to individually fit the M-dimer models into the sub-tomogram average and define a centroid for each that was used for measuring the distance and angles between dimers (Fig. 2c, e, f). The center to center spacing between the modeled dimers was 53.6 ± 0.03 Å (mean ± standard deviation, *n* = 8), 66.1 ± 0.04 Å (*n* = 8), and 79.5 ± 0.07 Å (*n* = 7). We measured the angles between dimers (as indicated in Fig. 2f) to be 82.5 ± 0.08 degrees (*n* = 6). The minor variations in distances and angles measured within the lattice may be due to factors such as imperfect model fitting or variations in lattice curvature within and between different segments. The range of diameters from the virion segments included in our sub-tomogram average of M was 103–182 nm, with a mean and standard deviation of 132 nm and 18 nm (Supplementary Fig. 4). The potential causes and implications of variation in the lattice spacing are detailed in the discussion section.

### Sub-tomogram averaging of a pair of F trimers

**Fig. 3:**
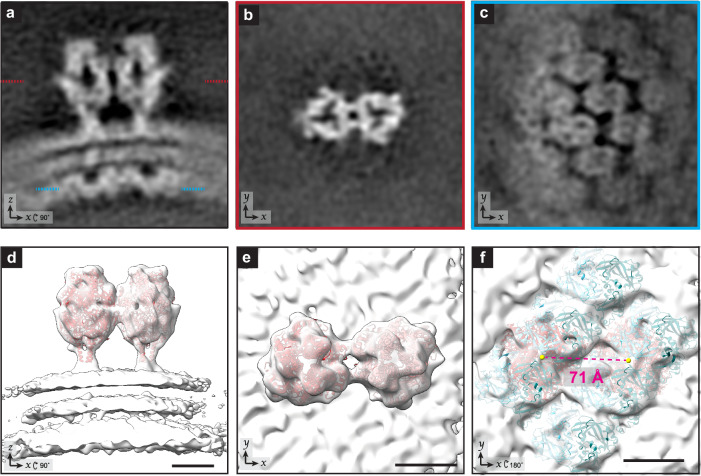
Sub-tomogram average of RSV F pair. **a** Slice from a sub-tomogram average of a pair of RSV F trimers on the viral envelope. The outer lipid layer, inner lipid layer, and M layer can be seen underneath. The z-position of the sections in **b** and **c** are indicated with red and cyan dashed lines respectively. **b** Slice from the sub-tomogram average from **a**, viewed looking towards the membrane from outside of the virus. The two trimers are arranged in an antiparallel fashion. **c** Slice from the sub-tomogram average from (**a**) showing the M layer. **d** Isosurface renderings of the sub-tomogram average in (**a**) with RSV F pre-fusion trimer models (PDB: 4JHW) fit in. **e** Rotated view of **d**, viewed looking towards the membrane from outside of the virus. **f** Isosurface renderings of the sub-tomogram average in (**a**) with models of RSV F (PDB: 4JHW, red) and models of M dimers (PDB: 4v23, dark blue and cyan) fit in. Viewed from the interior of the virus (180° rotation from **c**). A yellow sphere has been modeled in over the centroid of each F trimer. The center-to-center distance between the two fit F-trimers is shown in red. Density map in (**a**–**f**) is an average from n = 21,522 sub-tomograms. 5 nm scale bar for all panels.

To better understand the organization of F on the virion surface we determined a sub-tomogram average of an F trimer pair (Fig. 3, Supplementary Movie 2). The resulting average contained two trimeric structures that extended approximately 12 nm above the membrane surface, consistent with previously reported structures of prefusion F on virions (Fig. 3a, b)^7,9,15^. To further validate that the structures were RSV prefusion F homotrimers, we fit in a model from a previously determined crystal structure of RSV F prefusion trimers (PDB: 4JHW^59^) (Fig. 3d, e). The sub-tomogram average revealed that the F trimers were not positioned randomly with respect to each other on the virion but were positioned in an antiparallel manner with their respective vertices rotated 180° with respect to each other (Fig. 3b, e). Furthermore, the placement of an individual trimer was determined by its position within the trimer pair, where one of the vertices of the left most trimer was always pointed in an upward orientation. This positioning was validated by the model fitting (Fig. 3d, e). Although we cannot rule out that there may be other arrangements of the trimer clusters, we were unable to generate sub-tomogram averages in which the trimer pairs had other orientation parameters.

### M lattice coordination of F positioning

To define the relationship between M and the F glycoproteins on the virion surface, we examined the density corresponding to the M layer within the sub-tomogram average of the F trimer pairs. The lattice organization of the M layer was clearly preserved (Fig. 3a, c, Supplementary Movie 2). If the F pairs were randomly positioned with respect to the underlying M lattice, an average of the M lattice would not have been revealed. Instead, the pair of F trimers and M lattice were resolved within a single sub-tomogram average, which indicated that positioning of M and F are coordinated relative to each other.

We were unable to resolve the cytoplasmic tail of F within the sub-tomogram average. To better visualize the position of F relative to the M lattice we modeled both F-trimers (PDB: 4JHW) and M-dimers (PDB: 4v23) into the sub-tomogram average and placed a yellow sphere at the centroid of each F-trimer. When viewed from the viral interior (Fig. 3f) the two F trimers are centered over similar positions in the lattice between M-dimers. However, the center-to-center distance between the modeled F-trimers (Fig. 3f) was measured to be 71 Å compared to the 78 Å distance between two M dimers (Fig. 2f). This discrepancy could arise from the flexibility of the F-trimer stalk and cytoplasmic tails^59,60^. We also observed additional density in the sub-tomogram average connecting the two trimers (Fig. 3b, e). Though the resolution was not sufficient to confirm a direct interaction, the presence of connecting densities between the trimers would be consistent with the trimers having a closer center-to-center distance than the cytoplasmic tails.

### Sub-tomogram averaging higher-order organizations of F

**Fig. 4:**
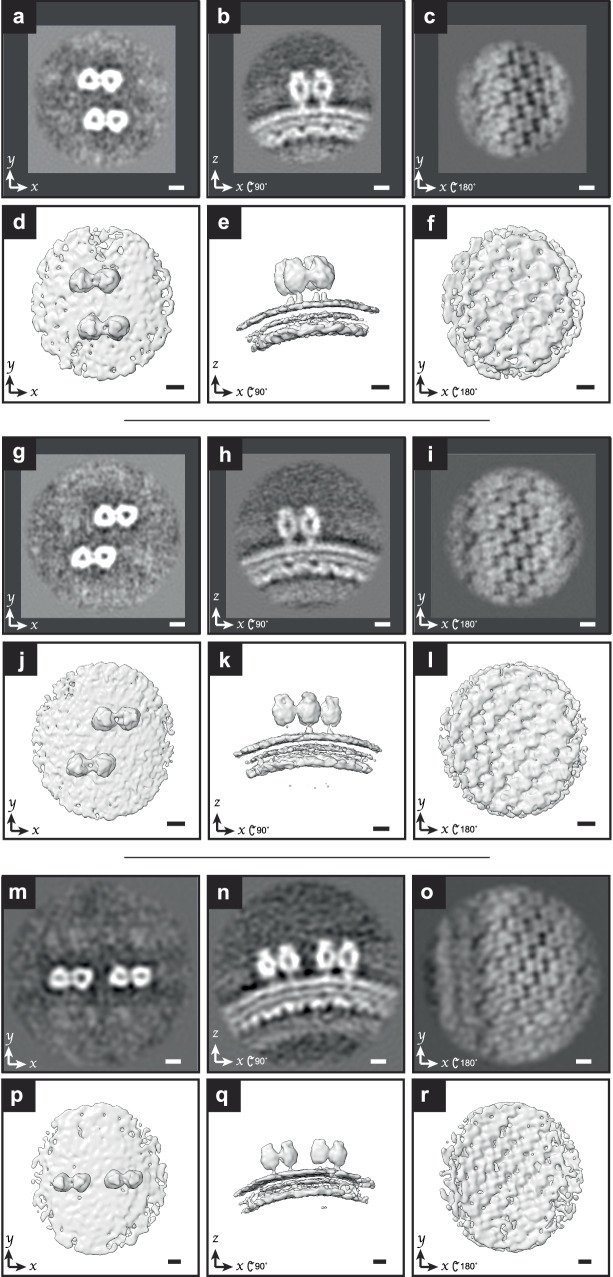
Sub-tomogram averages of representative RSV F pairs. **a** Single slice from sub-tomogram average of four RSV F trimers. **b** Slice from average in **a** viewed rotated −90° around x. **c** Slice from average in **a** rotated 180° around the x-axis (as viewed from virus interior) through the position of the M-lattice. **d**–**f** Isosurfaces of the sub-tomogram average as shown in **a**–**c** respectively. **g**–**i** Slices from a sub-tomogram average density map of a different arrangement of f-trimers than (**a**–**f**). **j**–**l** Isosurfaces of **g**–**i**. **m**–**o** Slices from a sub-tomogram average density map from a third arrangement of f-trimers. **p**–**r** Isosurfaces of **m**–**o**. Density map in **a**–**f** is an average from *n* = 1030 sub-tomograms, *n* = 1246 sub-tomograms for **g**–**l** and *n* = 1132 sub-tomograms for **m**–**r**. 5 nm scale bar for all panels.

The organization of F on virions was not limited to isolated pairs, but appeared to have higher order organization, such as the presence of rows (Fig. 1e). We used masked principal component analysis (PCA) classification in the processing software PEET^61^ to classify particles based upon areas outside of the central pair of F-trimers. Using this strategy we identified three distinct sub-tomogram averages, each with two pairs of trimers (Fig. 4, Supplementary Movies 3–5). Each average included between one thousand to thirteen hundred sub-tomograms. The conserved anti-parallel orientation of the trimers was observed in the three sub-tomogram averages. Furthermore, density connecting individual trimers and clear preservation of the M lattice beneath the F-pairs was present in all three averages.

## Discussion

Matrix proteins have been identified as central organizers of virus assembly in negative-strand RNA viruses^21–24^. RSV and other *Pneumoviridae* family members are closely related to viruses of the *Paramyxovidae* family, including measles virus and Newcastle disease virus (NDV) for which the organization of matrix has been determined^30,33^. For both viruses, sub-tomogram averaging was previously used to show that matrix is arranged in a lattice of dimers and the glycoproteins are similarly arranged in a lattice^30,33^. However, given the smaller size of RSV M (28 kDa) and the fact that it was initially crystallized as a monomer^27^, questions have remained about the higher-order organization of M in RSV. Our results from cryo-ET and sub-tomogram averaging of virions show that M is arranged in a locally ordered lattice of dimers with an apparent helical organization relative to the viral long axis. This is consistent with the organization previously reported by others^20^ and the organization in related viruses^30,33–35^. These findings support prior studies that found M dimerization is required for the assembly of filamentous VLPs^26^.

### Organization of RSV M

Our sub-tomogram average revealed a tightly packed lattice of protein associated with the interior of the RSV membrane. Model fitting of the RSV M crystal structure (PDB: 4v23^26^) confirmed that the lattice is composed of RSV M dimers and allowed for identification of residues at the dimer-dimer interfaces. (Fig. 2, Supplementary Movie 1, Supplementary Fig. 5). The repeating M dimer lattice has an average spacing of 5.4 nm, 6.6 nm, and 8.0 nm (Fig. 2f). The orientation and spacing of this lattice is similar to the packing of the crystallized form of M (PDB: 4v23) that had an asymmetrical unit consisting of an M dimer with unit cell dimensions of 52.3 Å, 65.9 Å, and 79.2 Å. The orientation of RSV M relative to the membrane in the sub-tomogram average is consistent with previous structural analysis of RSV M^27^. Conley et al. have recently reported a lower resolution sub-tomogram average of the RSV M lattice with a similar orientation and organization to our results presented here^20^. Our sub-tomogram average also reveals density linking M dimers that does not appear attributable to M protein (Supplementary Fig. 3). We were not able to identify the source of the density at our current resolution, but it is an intriguing target for further biochemical and structural studies of the matrix lattice. Sub-tomogram averaging of the mature matrix lattice from HIV-1 revealed density in PI(4,5)P2 binding pocket^36^. However, RSV M does not have a known lipid binding pocket and the connecting density in our sub-tomogram average is on the cytoplasmic side of the lattice.

Our results provide further evidence that lattice organization of matrix is broadly conserved across several negative-strand RNA virus families. In many cases the structure of matrix itself is conserved, crystal structures of RSV and NDV matrix proteins have an RMSD of only 3 Å^33^. Lattice organizations of matrix proteins have been shown for a number of viruses with diverse virion morphologies including NDV^33^, measles virus^62^, rabies virus^63^, vesicular stomatitis virus (VSV)^32^, Ebola and Marburg viruses^34^, influenza A virus^35^ and others (Supplementary Table 1). VSV and rabies virus have bullet-shaped virions approximately 70 nm in diameter and 200 nm in length with relatively low variation (~50 nm) in either dimension^32,63^. Measles and NDV have ellipsoidal virions that vary in shape from spherical to near filamentous. In particular, the dimensions of measles virions can range from 50 nm to >500 nm^30,31^. Ebola and Marburg have filamentous virions that vary in diameter by ~20 nm, with lengths extending to many micrometers^34^, while influenza A forms both spherical and filamentous virions^35,64^. The organization of the lattices formed by viral matrix proteins is also quite variable. The matrix lattices of Measles and NDV have four-fold symmetry, VSV and rabies virus matrix are helical assemblies, while other lattices formed by matrix retain two-fold symmetry with more limited helical character. The apparent helical pitch among those with two-fold and/or helical symmetry varies from close to zero for Marburg and rabies viruses^34,63^ to the 48° reported here for RSV.

RSV has filamentous virions that can vary in length by several micrometers and in diameter by more than two-fold (Supplementary Fig. 4)^7,15,20^. Arrays of M can be seen lining the membrane of filamentous virions with a range of diameters, as well as along flatter segments of irregular viruses. However, regions of ordered M are often not present in areas of high membrane curvature such as at the ends of filamentous particles, and at bends or branch points in irregularly shaped virions^7,8,15^. Different curvatures of the lattice would require lattice flexibility supported by altered spacing between subunits. Flexibility in the M lattice could be achieved with plasticity at the dimer interface, which has been demonstrated through comparison of the two M dimer crystal structures^26^. There may be additional flexibility in dimer-dimer organization. The presence of gaps or irregularities in the lattice could be an additional mechanism for achieving a range of local curvatures, but also suggests a limitation to the flexibility of the intact lattice. Irregularities and gaps have been observed in the matrix protein lattice of other viruses^33,34,65,66^. Our ability to resolve a high-resolution structure with consistent spacing between dimers indicates that the reported lattice spacing is at least prominent in virions. However, the particle positions included in our final sub-tomogram average contain gaps and do not include all possible M lattice positions from the virions (Fig. 2a). These gaps could be due to technical limitations of the particle picking and template matching approaches used but may also include areas of absent or differentially organized M-dimers. Further study will be necessary to determine the extent of flexibility of the M lattice in RSV virions. Using the 48° apparent helical angle measured in our sub-tomogram average and subtracting 14 nm from the measured diameters (Supplementary Fig. 4) to account for the spacing between the viral membrane and M lattice, the range for the radius of curvature at the level of the M lattice was calculated to be 99.4 nm to 187.6 nm (Supplementary Fig. 4). For comparison with other filamentous virions, the radius of curvature of Marburg virus VP40 was reported to be ~45 nm^34^. Though a radius of curvature was not reported for either, Influenza A M1 radii ranges from 18 to 29 nm^35^ and VSV M1 and M2 radii is ~18–22 nm^67^.

One potential mechanism for driving the diversity of virion morphology and lattice structure is the contribution of additional structural proteins. Ebola VP40 and measles virus M are sufficient to assemble filamentous VLPs^68,69^. For Ebola, the expression of additional structural proteins alters both the VLP diameter and helical pitch of the VP40 lattice^34^. Nipah virus M, F, and to a lesser extent G are all independently capable of budding VLPs^70,71^. Influenza matrix (M1) associates with but is not required for VLP formation driven by Hemagglutinin and Neuraminidase^72^. Purified RSV M can assemble into a uniform helical filament in the presence of specific lipids^73^, but requires expression of P and the F cytoplasmic tail for assembly of filamentous particles in cells^37,38^. Many viral matrix proteins have also been shown to interact with and/or coordinate the positioning of the nucleoprotein^31–33,35,62,63^. The potential role of additional RSV structural proteins in determining viral morphology will require further studies.

### Organization of RSV F

RSV F is a class I fusion protein that may also support cell attachment. Though direct interaction between RSV M and F has not been shown, expression of F is required for recruitment of M to lipid rafts and similar functional interactions have been shown in related viruses^74–78^. The cytoplasmic tail of RSV F is essential for filamentous VLP assembly^75^. RSV F is present on virions in a prefusion state and undergoes an irreversible conformational change during membrane fusion into a post-fusion state. The transition to the post-fusion state can be induced by other factors, as well, such as heat^8,62^. Structures of the RSV F homotrimer have been solved in both the pre- and post-fusion states, though these structures lack the transmembrane segment and cytoplasmic tail^14,59^. Sub-tomogram averaging of RSV F on virions has been used to distinguish between the pre- and post- fusion states of large numbers of individual trimers^9,15^.

Our sub-tomogram average contains a pair of potentially interacting F trimers (Fig. 3, Supplementary Movie 2). Fitting of the prefusion-F trimer crystal structure confirmed that the paired densities on the virus surface visible in the tomograms are F trimers. The trimers are oriented relative to one another within a pair and the pairs share a common orientation relative to the virus. This was also seen in our sub-tomogram averages with four F trimers each (Fig. 4, Supplementary Movies 3–5). While pairing of trimers could be explained by local interactions between trimers, the common orientation of pairs requires a model including additional regulation of F positioning on the virion. Coordination with the M-lattice is one possibility that is also consistent with the observation that the pairs of trimers are positioned in preferred positions relative to one another (Fig. 4). Our data support a model in which each F-trimer is positioned over an equivalent point relative to an underlying M-dimer. This may occur at an M dimer-dimer interface as indicated by the modeling in Fig. 3f, however given the potential flexibility of the F stalk and cytoplasmic tail, further work is necessary to determine the precise position. An interaction between the F-cytoplasmic tail and the M lattice would be one possible mechanism. This model is further supported by the necessity of the F-cytoplasmic tail to assemble filamentous virus-like particles^75,76^.

Applying this model to interpret the distribution of F on the viral surface, F trimers can occupy positions in rows roughly perpendicular to the viral long axis with no unoccupied positions in between (Fig. 4m). The formation of pairs pulls the F trimer heads closer together causing them to appear unevenly spaced despite their regular spacing relative to the M lattice. Unoccupied positions in these rows further contribute to the appearance of uneven spacing. The sub-tomogram averages in Fig. 4a, g represent pairs of F trimers with two unoccupied rows in between. Variation in the gaps between rows means that though the positions of the trimers are regulated by the underlying helical-like M lattice, it may not be possible to follow a single row of F-trimers over an entire helical turn around the virion.

Positioning of F trimer pairs through coordination with the M lattice would support many organizations of F trimer pairs in addition to those shown in Fig. 4. Our classification and processing in this work was focused on resolving a few prominent states rather than a characterization of all possible organizations present. It must also be taken into consideration that the sub-tomogram averages from this work do not exhaustively capture all F-trimers on the virion surface. Isolated F-trimers are seen on the virions in our tomograms (Fig. 1e) and in previous work. The further characterization of F-trimer organization and potential coordination of single F-trimers with the underlying matrix lattice will be pursued in future work.

The presence of F pairs raises many interesting questions as to whether this coordinated arrangement is functionally important for the virus. Close packing of F could affect antibody and/or receptor binding or it may promote fusion since clustering of class I fusion proteins is known to occur during membrane fusion^79–81^. Pairing of F could also affect the stability of the F prefusion state^82,83^. It is possible that the ordered rows of F pairs on the virions are one transient arrangement that exists over a continuum of intermediate F trimer states. This overall organization of F trimers may evolve over time due to fluidity in the viral membrane and underlying M lattice, possible associations with secreted or membrane-associated G, or interaction with receptor and/or other cellular proteins. Furthermore, it is known that class I viral fusion proteins exist in a ‘metastable’ prefusion structural state, and that upon ‘triggering,’ which is caused by cascading interactions with another glycoprotein and/or cellular receptor and/or co-receptor, the protein undergoes an irreversible conformational change to the post-fusion state^14,59,83^.

Until recently there has been limited structural information about the coordinated organization of fusion or attachment glycoproteins in either purified protein preparations or on intact virus particles. Negative stain TEM and cryo-ET of purified HPIV3 illustrated that HN, in the ‘heads down’ conformation, formed regular arrays on virions and that F was absent in these regions^84^. However, no lattice-like arrangement of pre- or post-fusion F alone or F colocalized with HN in the ‘heads up’ orientation was observed on the virions, indicating the transient nature of these glycoprotein arrangements. We have previously demonstrated that measles virus F is presented in an organized lattice with four-fold symmetry coordinated by an underlying lattice of M^30^. Cryo-EM of purified prefusion F-trimers from Nipah virus, Hendra virus, and Langya virus showed all three form a dimer-of-trimers in solution^85^. In addition, Xu et al. demonstrated by X-ray crystallography and EM that prefusion stabilized Nipah virus F trimers could form a semi-stable hexamer of trimers both in vitro and on VLPs^82^. This validates the requirement for preserving and locking these classes of proteins into a single conformational state for intermediate to high-resolution structural analyses^59,82,86^. Though we did not observe hexagonal organization of RSV F on virions in this study, hexagonal lattice packing of RSV F on intact, filamentous virions has been reported for vaccine-candidate strains that were engineered with stabilizing mutations in F and reduced G levels^62^. This further supports the ability of RSV F prefusion trimers to assemble into varied states of higher-order oligomerization, potentially in a transient or condition dependent manner. Arrays of RSV F were also observed on irregular particles by Conley et al.^20^. In kind, the fusion protein of influenza type C, HEF1, was shown to assemble as hexagonal arrays^87^. The inter-trimer interactions of F trimers within the Nipah F hexamers and HEF1 hexagonal array are similar to what we report for RSV F dimers (Fig. 3b, e) providing evidence that the assembly of RSV F into pairs or hexamers may occur through interactions conserved between a number of viral class I fusion proteins.

Our sub-tomogram averaging from whole-cell cryo-ET of RSV virions shows that M is present as a tightly packed lattice of dimers with a helical-like arrangement in filamentous virions. Further, the RSV F glycoprotein is not randomly distributed on the virion, but frequently occurs as pairs of trimers and in specific positions consistent with coordination of F positioning by the underlying M lattice. Future studies will be needed to determine the nature of the structural contacts present between the M lattice and F as well as conditions under which RSV prefusion F trimers oligomerize and the functional role of these arrangements.

## Methods

### Cell culture and infection on TEM grids

BEAS-2B cells (ATCC CRL-9609) were cultured and maintained at 37 °C with 5% CO_2_ in RPMI-1640 (Thermo Fisher Scientific) media supplemented with 10% fetal bovine serum (FBS, Hyclone) and 1× antibiotic antimycotic solution (100 units/mL penicillin, 0.1 mg/ml streptomycin and 0.25 µg/ml amphotericin B, Thermo Fisher Scientific). Vero cells (ATCC CCL-81) were maintained in DMEM (Thermo Fisher Scientific) supplemented with 10% FBS and 1× antibiotic antimycotic solution. Cells were released for passaging or seeding onto EM grids using 0.25% trypsin (Thermo Fisher Scientific). Cell counting was done using a hemacytometer following trypan blue staining.

### Respiratory syncytial virus and infection on TEM grids

The RSV strain rA2-mK+ strain was used for all experiments. RSV rA2-mK+ shares the same growth kinetics as its parent strain, A2, and expresses the fluorescent protein mKate2 during replication due to insertion of this gene at the 3’ end of the genome^88^. Viral titer was determined in Vero cells using a fluorescent focal unit (FFU) assay^7^. Vero cells were seeded in 96-well plates at a density of 2 × 10^−4^ cells/well. Cells were spinoculated with serial 10-fold dilutions of the virus stock at 1900 rcf for 30 min at 4 °C. The inoculum was removed and cells were overlaid with 0.75% methylcellulose in DMEM. Fluorescent foci were counted 48 ho post infection. The average of three technical replicates in the 96-well plate was used as the final titer.

To provide extra support for cell growth^89^, 5–6 nm of carbon was evaporated onto Quantifoil Au 200 R2/1 or R2/2 grids (Quantifoil Micro Tools GmbH). Grids were then glow discharged and incubated in supplemented RPMI-1640 overnight in the cell incubator. Two to four grids were placed in a 35 mm glass bottom dish (MatTek Corp) and 7.5 × 10^4^ BEAS-2B cells were added in 2 ml of media. After overnight incubation (15–17 h) the media was removed, and the cells were infected with RSV rA2-mK+ at a multiplicity of infection (MOI) of 10. The grids were plunge frozen using a Cryoplunge 3 (Gatan, Pleasanton, CA) 24 h post infection. BSA gold tracer (10 nm, Aurion) was added immediately prior to freezing as a fiducial marker for tilt-series alignment.

### Cryo-electron microscopy and tomography

CryoEM images were collected on a Titan Krios 300 kV electron microscope (Thermo Fisher Scientific) equipped with a Gatan K3 direct-electron detector and BioQuantum energy filter (20 eV slit width, Gatan, Pleasanton, CA) (Supplementary Table 2). Tilt-series were collected with a dose symmetric scheme^90^ from 0° in 3° increments, groups of two, from −60° to +60° with a total dose of ~100 e/Å^2^ using SerialEM^91^. The unbinned pixel size was 1.693 Å and the nominal defocus was −2 to −6 μm in 0.25 µm steps.

Frames were aligned with MotionCor2^92^. Tilt-series were aligned and processed in IMOD^93^ including CTF correction by phase flipping and dose-weighting. Tomograms were reconstructed in IMOD using weighted back-projection from tilt-series binned by six, four, and two. Bin six tomograms were processed with IsoNet^94^ for denoising and missing wedge correction to enhance visualization of membrane and matrix. IsoNet corrected tomograms were examined to select 27 tomograms with well-preserved virus segments for sub-tomogram averaging. Segmentation (M) and manual particle picking (F) were done on IsoNet processed tomograms. Select tomograms were denoised with cryoCARE^95^ at bin four (from weighted back-projection volumes, no IsoNet processing) for improved visual analysis as shown in Fig. 1. The density profile plot in Fig. 1 was generated in Fiji 1.53^96^.

### Sub-tomogram averaging

Sub-tomogram averaging was done in PEET 1.15.0^61,97^ and Relion 4.0^98,99^. Tomograms reconstructed with a binning of six were processed with IsoNet^94^ to enhance visualization of membrane and matrix particularly in the missing-wedge. Thirty-eight filamentous sections from 33 virions present in 27 tomograms were oriented as in Fig. 1 with the long axis vertical and flat in the image plane. The matrix layer was manually segmented in 3dmod^100^ and the segmentation was used to seed a regularly spaced grid of points with a minimum spacing of five pixels, which corresponded to 51 Å. PEET spikeInit was used to generate initial orientations for the particles. The tomograms were divided into three batches for initial alignment/template-matching in PEET using the same reference generated from a single particle selected from one of the tomograms. The seeded points were aligned to this initial reference allowing sufficient translation to overlap with the search range of neighboring points and duplicate points (within five pixels, 51 Å) were discarded. See Supplementary Table 3 for starting and retained particle number and other details for each iteration of alignment.

The aligned points were updated and scaled for tomograms reconstructed at bin four and the tomograms were divided into two batches. The final average from one of the bin six alignments was scaled to a bin four-pixel size and lowpass filtered to 40 Å with EMAN2^101^ and used as the initial reference for the bin four alignments. Duplicate points (within eight pixels, 54 Å) were discarded. The FSC at 0.5 for the two bin four maps was calculated to be 17 Å using the PEET program simpleFSC. The aligned points were updated for bin two tomograms and the final average from one of the bin four alignments was lowpass filtered to 25 Å and used as the initial reference for a round of alignment at bin two. Duplicate points (within 14 pixels, 47 Å) were removed.

3dmod programs imodtrans and MOTL2Relion were used to convert the points and orientations to Relion conventions necessary to build a coordinates star file for use in Relion 4.0. The tomograms used for segmentation and PEET were originally reconstructed with x-axis tilt correction, so the models and particle orientations were also rotated around X (inverse of angle applied to the tomogram) to correct for the x-axis tilt, which was not applied to the tomograms by Relion.

An average using the refined positions and orientations imported from PEET from all 27 tomograms was reconstructed at bin one in Relion without alignment. The bin one averages were used for CTF refinement and frame alignment. Bin two pseudo-sub-tomograms were generated from the refined tilt-series and used for particle alignment in Relion. The aligned particles were reconstructed at bin one and used for another round of CTF refinement and frame alignment. Following the bin two alignment, the particles were subjected to geometric cleaning. Any particle that did not meet the following requirements was discarded: at least three neighboring particles must be within 128 pixels in X, Y and ten pixels in Z (relative to the particle orientation) and must have a y-axis angle within 15° of the particle y-axis angle. Starparser^102^ was used to extract particle positions and orientations from the Relion star files and a custom script was created to identify particles that did not meet the described requirements. Following the cleaning, a bin one reconstruction was generated and used for CTF refinement and frame alignment. Bin one pseudo-sub-tomograms were generated from the refined tilt-series and used for alignment at bin one. A final round of CTF refinement and tomo frame alignment was done before reconstruction of the final average. The final resolution of the map of the M lattice was 4.6 Å based on the 0.143 Fourier shell correlation (FSC) criterion (Supplementary Fig. 6). Visualization of model points over the tomographic slice was done in UCSF Chimera 1.15 using the Place Object plugin^103^.

Sub-tomogram averaging of the F pair started with manual selection of F pairs from five bin six tomograms and spikeInit was used to define initial orientations of the particles. The points were aligned in PEET using a single particle initial reference and duplicate particles within four pixels (41 Å) were removed. The final average was lowpass filtered to 50 Å. See Supplementary Table 4 for starting and retained particle number and other details for each iteration of alignment. For initial alignment/template-matching from all 27 tomograms in PEET the initial points used for matrix were shifted by 14 pixels (~14 nm) in Z, positioning them outside the viral membrane and in the proximity of F. The lowpass filtered average from the manually selected particles was used as an initial reference for a round of alignment at bin six. The tomograms were split into three batches for the bin six alignment as was done for matrix. PCA classification was used to classify the aligned particles and classes that did not resemble a pair of F trimers were excluded from further processing. The remaining particles were used for the next round of alignment done at bin four in PEET. Bin four alignment was done in two batches of tomograms using one of the final bin six aligned averages lowpass filtered to 40 Å as an initial reference. PCA classification was again used to remove particles in classes that did not resemble a pair of F trimers. The remaining particles were used for the next round of alignment at bin two in PEET using one of the bin four aligned averages lowpass filtered to 30 Å as an initial reference. A third round of PCA classification was done following the bin two alignment and particles in classes that did not resemble a pair of F trimers were excluded from further processing.

Relion coordinate star files were generated as previously described for matrix and a bin one reconstruction was generated from the imported positions and orientations for CTF refinement and frame alignment. Bin two pseudo-sub-tomograms were generated from the refined tilt-series and used for alignment in Relion at bin two. The aligned particles were reconstructed at bin one and used for another round of CTF refinement and frame alignment. Bin one pseudo-sub-tomograms were generated from the refined tilt-series and used for alignment at bin one. The final resolution of the map of the pair of F trimers was 14 Å based on the 0.143 Fourier shell correlation (FSC) criterion (Supplementary Fig. 7).

To generate averages of the multiple F pairs the final PEET bin two F-pair averages were reconstructed with larger box sizes to include more of the surrounding area. Masks were generated to perform PCA classification of the particles based on density in areas above, below, and to either side of the F-pair (Supplementary Fig. 8). Two masks in different positions above the pair were used with masks in the corresponding position below the F-pair. A mask was also generated to the left and right for six PCA masks in total. PCA was performed using each mask independently on the particles from each of the two sets of tomograms (Supplementary Table 5). Particles from classes that had a second pair of F-trimers in addition to the central pair were kept for further processing. Each class was independently reconstructed in PEET. The averages from equivalent organizations of trimers were aligned and averaged in PEET recentering the average on all four trimers. For example, classes with an F-pair on the left of the original pair from each set of tomograms were averaged with classes with an F-pair on the right of the original pair from each set of tomograms and the center of the average was shifted to be in between the two pairs. These averaged classes were lowpass filtered to 50 Å and used as an initial reference for a round of alignment in PEET using the individual particles in the classes. The particle positions were roughly shifted to match the centering of the initial reference prior to alignment using modifyMotiveList. A final round of PCA classification was done and particles in classes that did not resemble two pairs of F-trimers were excluded from further processing. See Supplementary Table 6 for starting and retained particle number and other details for each iteration of alignment.

The coordinates and Euler angles were converted and assembled into Relion star files as for matrix and the single F-pair. Bin two pseudo-sub-tomograms were generated from the refined tilt-series and used for alignment in Relion at bin two. CTF refinement was done after bin two alignment. The final resolution of the maps multiple F trimer pairs in Fig. 4a, g, and m were 30 Å, 32 Å, and 36 Å, respectively, based on the 0.5 Fourier shell correlation (FSC) criterion (Supplementary Fig. 9). A schematic diagram of the sub-tomogram averaging workflows is shown in Supplementary Fig. 10.

### Reporting summary

Further information on research design is available in the Nature Portfolio Reporting Summary linked to this article.

### Supplementary information

Supplementary Information

Description of Additional Supplementary Files

Supplementary Movie 1

Supplementary Movie 2

Supplementary Movie 3

Supplementary Movie 4

Supplementary Movie 5

Reporting Summary

### Source data

Source Data

## Data Availability

The cryo-EM volumes from sub-tomogram averaging in this study have been deposited in the Electron Microscopy Data Bank (www.emdatabank.org) under the following accession numbers: 44965 (M lattice, Fig. 2), 44966 (F trimer pair, Fig. 3), 44968 (F trimer pairs, Fig. 4a), 44969 (F trimer pairs, Fig. 4g), 44971 (F trimer pairs, Fig. 4m). Source data are provided with this paper. All relevant data are available from the corresponding author upon request. Source data are provided with this paper.
