# Supplementary material for: Assembly of respiratory syncytial virus matrix protein lattice and its coordination with fusion glycoprotein trimers
Source: Nat Commun. 2024 Jul 14;15:5923. doi: 10.1038/s41467-024-50162-x (PMC11247094; doi:10.1038/s41467-024-50162-x)
Supplement: Supplementary file 1 — Supplementary Information [file 41467_2024_50162_MOESM1_ESM.pdf]

Assembly of respiratory syncytial virus matrix protein lattice and its coordination with fusion glycoprotein trimers

Sibert *et al.*

SUPPLEMENTARY INFORMATION.

SUPPLEMENTARY FIGURES, LEGENDS FOR SUPPLEMENTARY FIGURES, AND SUPPLEMENTARY TABLES.

SUPPLEMENTARY REFERENCES.

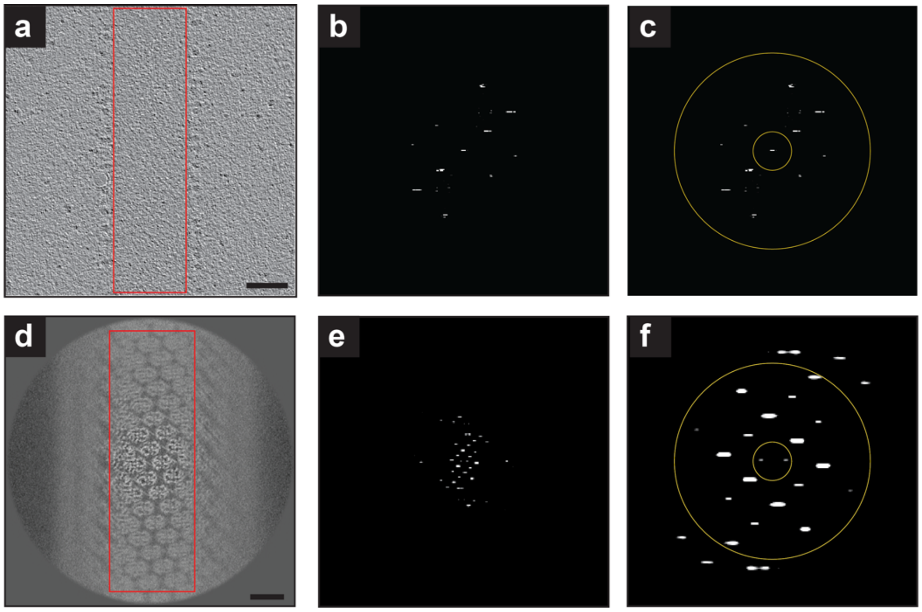

Supplementary Figure 1. FFT analysis of tomographic slice and M sub-tomogram average. a Single z-slice from a tomogram reconstructed at bin4 (6.76 Å/px) with the RSV filament oriented vertically in Y and with the M lattice flat in XY. The area in the red box was used for FFT analysis. **b** FFT amplitude rendering of the red box in (a) with levels adjusted to show brightest peaks. **c** Same image as (b) with yellow circles drawn at 10 nm^{-1} and 2 nm^{-1}. **d** Single z-slice from matrix sub-tomogram average (1.693 Å/px). The area in the red box was used for FFT analysis. **e** FFT amplitude rendering of the red box in (d) with levels adjusted to show brightest peaks. **f** Image in (e) cropped and enlarged to match to scale of (b, c) with yellow circles drawn at 10 nm^{-1} and 2 nm^{-1}. 50 nm scale bar in (a); 10 nm scale bar in (b).

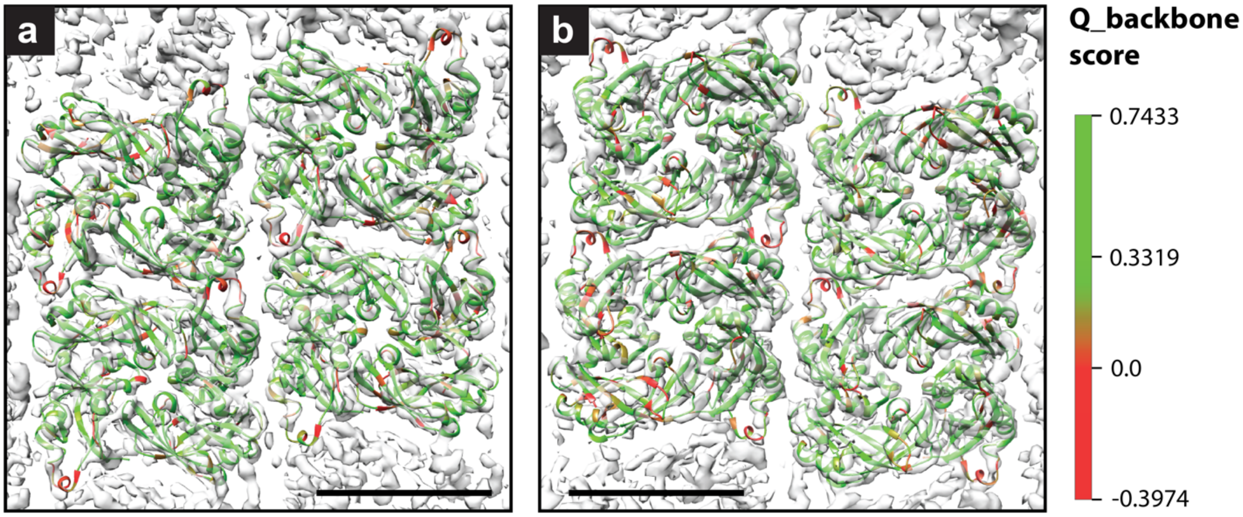

Supplementary Figure 2. Q-score analysis of cryo-EM map of M lattice with PDB:4v23. Copies of the atomic model from PDB:4v23 were fit into the density corresponding to the central four dimers of the cryo-EM density map of the M lattice using UCSF chimera. Q-scores were calculated using the MapQ plugin and visualized by coloring the ribbons according to the Q_backbone score. All residues with a Q-score above the expected Q-score @ 4.6 Å (0.3319) are colored in green, residues with lower values are colored as indicated in the scale bar on the right. The minimum (-0.3974) and maximum (0.7433) values displayed on the scale are the minimum and maximum Q_backbone scores present in the map/model. Source data are provided as a Source Data file. **a** Density map with fit models as viewed from the membrane toward the virion interior. **b** Panel (a) rotated 180° around the x-axis as viewed from the virion interior.

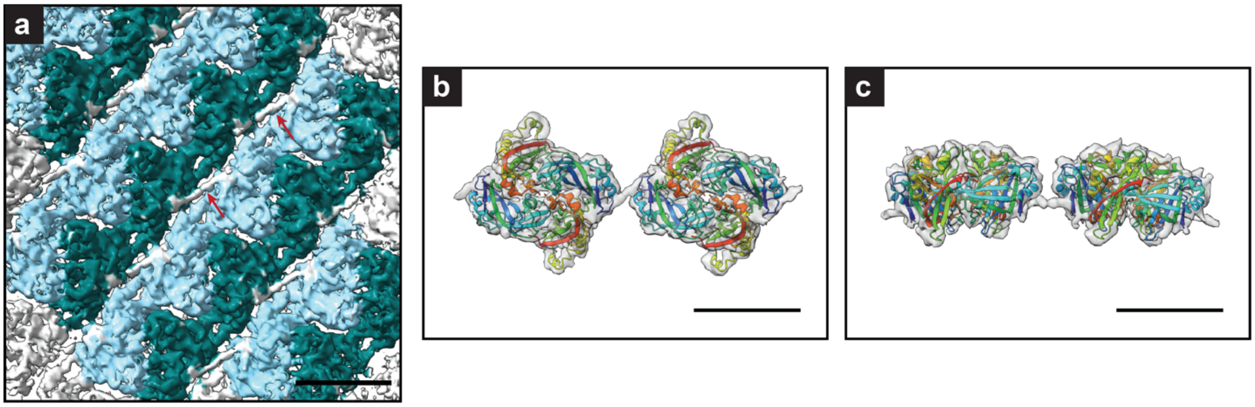

Supplementary Figure 3. Density connecting dimers in M sub-tomogram average.

a View of matrix sub-tomogram average from virus interior. Bi-colored copies of PDB: 4v23 were fit into the model as in Fig. 1 and all surfaces within 4.0 Å of the models were colored to match the nearest atoms. Densities in white, such as the densities between two dimers indicated with red arrows, represent areas greater than 4 Å from any atoms in the fit models. **b** Two of the central dimers from (a) are shown in the same orientation with surrounding density subtracted. Each monomer of PDB: 4v23 fit in is rainbow colored with the N-terminus in blue and C-terminus in red. **c** The surface and models in (b) rotated 90º with the membrane proximal side facing upwards. All scale bars are 5 nm.

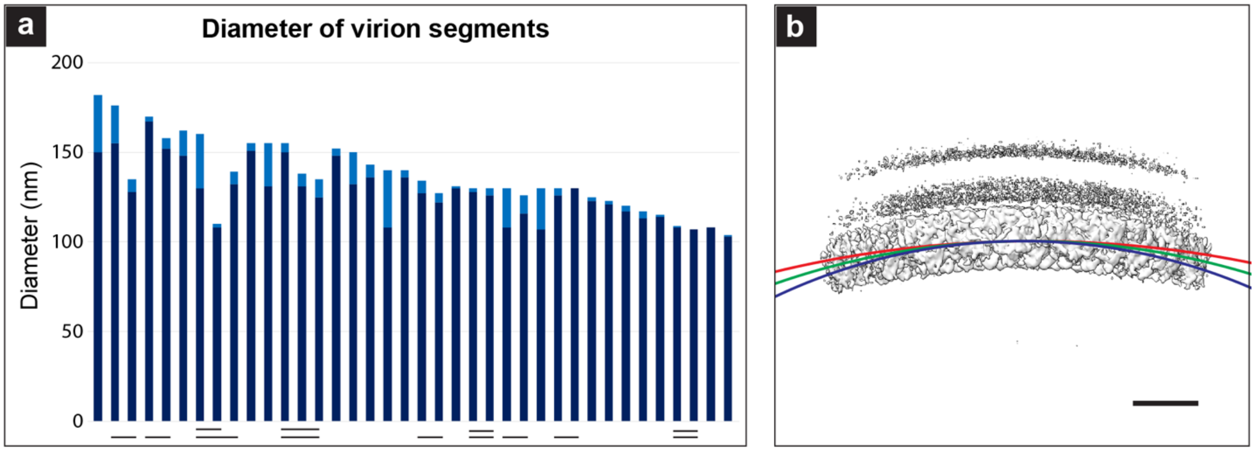

Supplementary Figure 4. Diameter of RSV virion segments used for sub-tomogram averaging. a Measurements of the virion diameter (membrane-to-membrane) near each end of the virion segment are graphed with the dark blue and light blue bars representing the smaller and larger diameter of the segment respectively. Individual straight segments from the same virion (connected by a bend or branch in the filament) are indicated by the presence of a line immediately beneath the bars in the graph. Individual segments from the same tomogram are indicated by a line on the bottom row of the graph. Segments from the same virion and tomogram will have two lines beneath the bars. Source data are provided as a Source Data file. **b** Arcs with curvature equivalent to circles with the measured minimum, maximum, and average virion diameter less 14 nm to account for the spacing between the membrane and M lattice are overlaid onto the M lattice sub-tomogram average. Red - 168 nm; Green - 118 nm; Blue - 89 nm. 5 nm scale bar in (b).

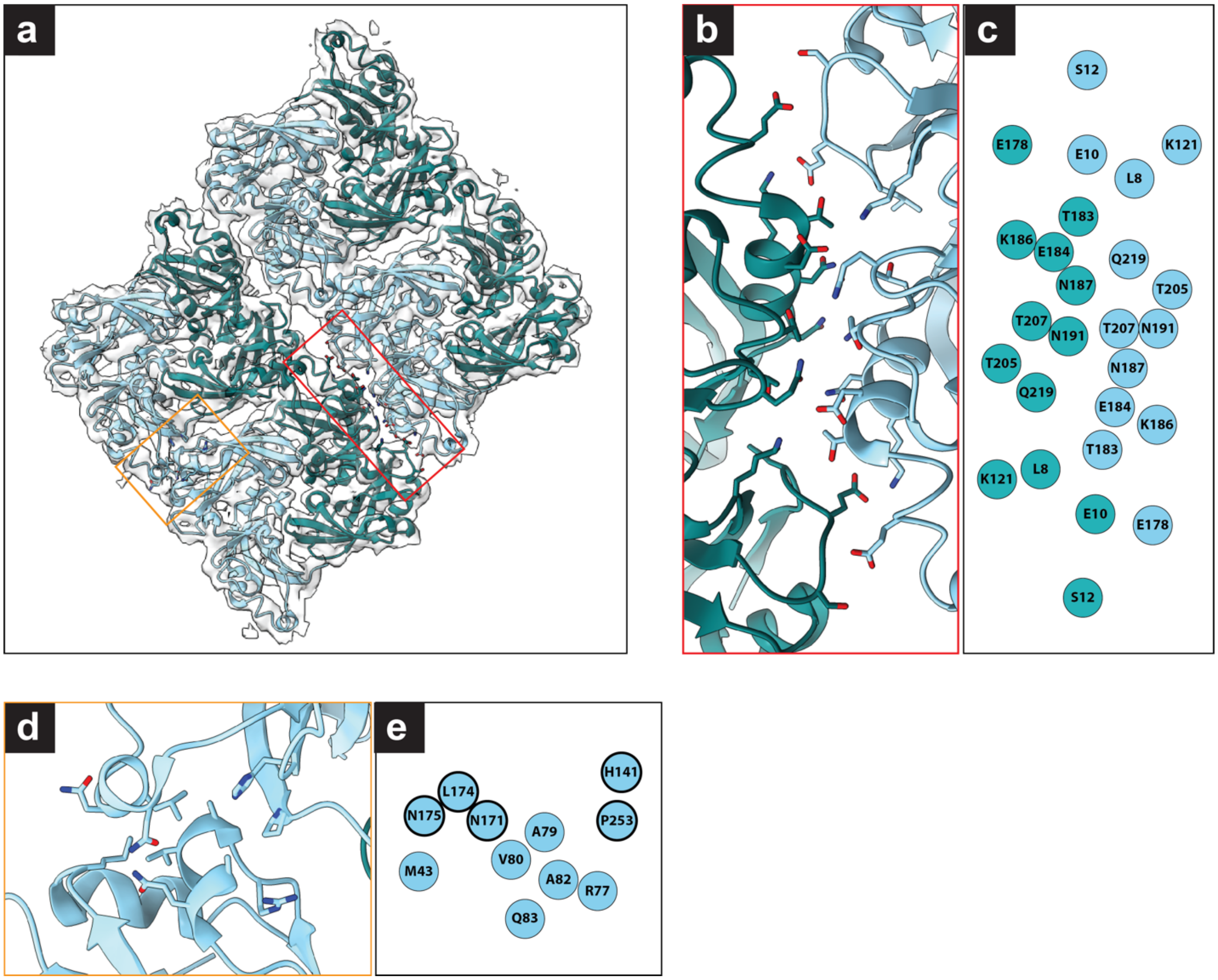

Supplementary Figure 5. Dimer-dimer interface residues. **a** Density map of M lattice with PDB:4v23 fit in, density further than 3.0 Å from the four fit dimers has been removed. Dimer-dimer interfaces are indicated with red and yellow boxes. **b** Dimer-dimer interface boxed in red in (a) with interface residues, defined by at least 15 Å^2 of buried solvent accessible surface, shown as sticks. **c** Cartoon diagram of interface residues in (b) showing the amino acid type and position. **d** As in (b) for the dimer-dimer interface boxed in yellow in (a). **e** As in (c) for the residues shown in (d).

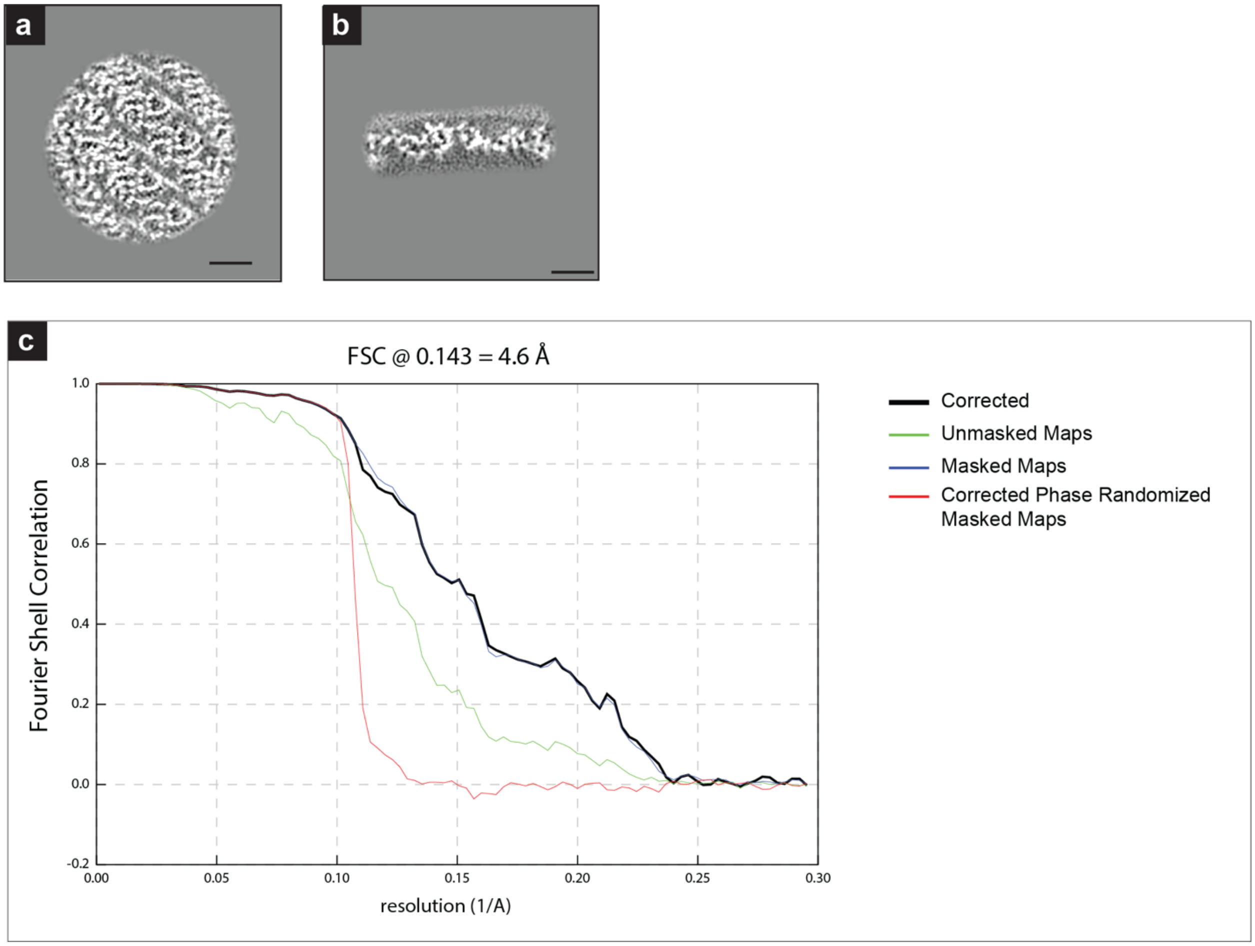

Supplementary Figure 6. Analysis of the sub-tomogram average of RSV M lattice.

a, b Masked slices from sub-tomogram average containing half of the particles used in the final average of RSV M. **c** Fourier shell correlation plot generated from Relion. Resolution is reported using FSC 0.143 of the masked corrected plot (black line). 5 nm scale bar in (a), (b).

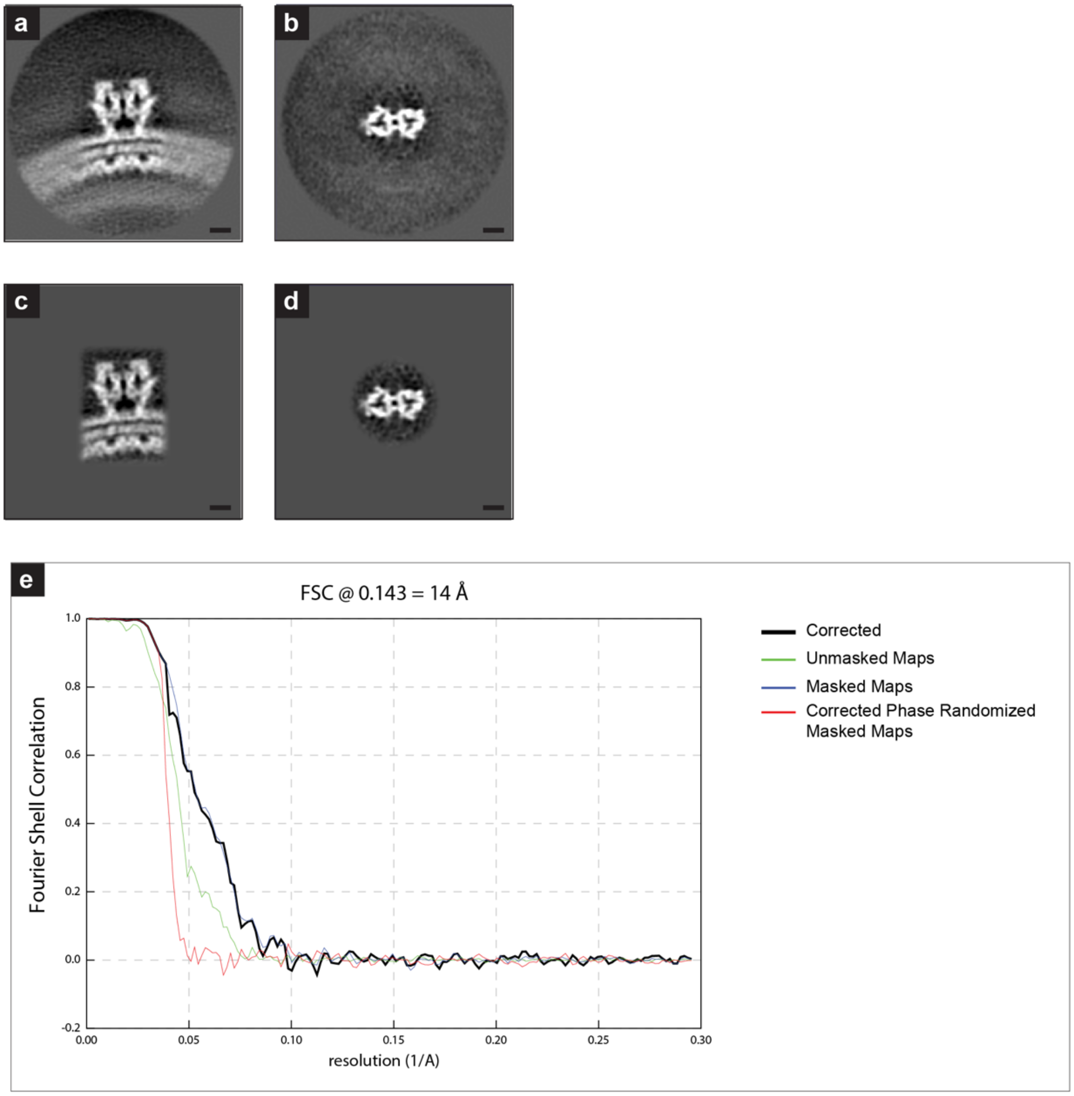

Supplementary Figure 7 | Analysis of the sub-tomogram average of the RSV F pair. a, b Slices from unmasked sub-tomogram average of the RSV F pair. **c, d** As in (a) with a soft-edged mask applied around the pair of F-trimers. **e** Fourier shell correlation plot generated from Relion. Resolution is reported using FSC 0.143 of the masked corrected plot (black line). 5 nm scale bar in (a), (b), (c), (d).

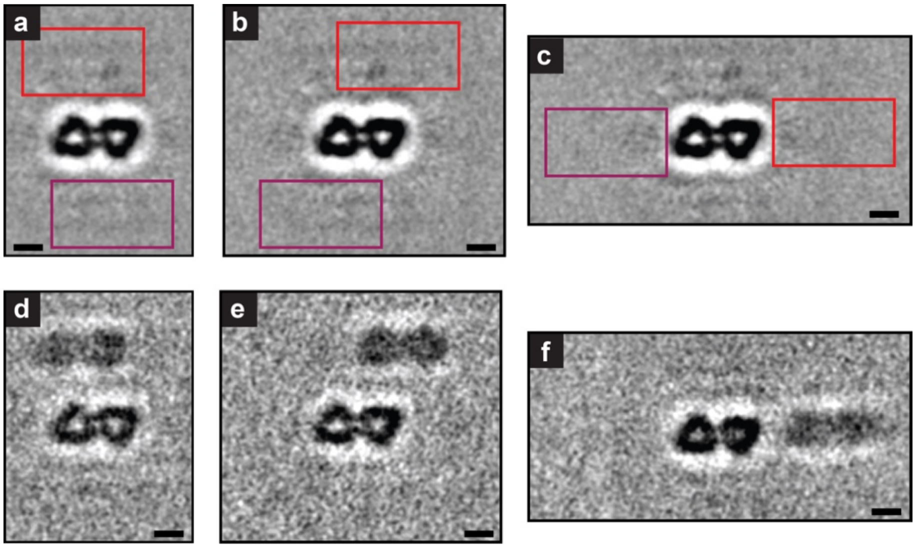

Supplementary Figure 8. PCA mask positions and class averages for multiple pairs of F-trimers. a, b, c Single slice from sub-tomogram averages of a pair of F-trimers after bin2 alignment in PEET (14 tomograms, 11,428 particles) reconstructed with box sizes of 96,128, 96 (x, y, z) in pixels; 144,128, 96 pixels and 196, 96, 96 pixels respectively. Two separate masked PCA classifications were performed for each average with the mask positions indicated by the red and maroon boxes. **d, e, f** Selected classes following PCA classification of (a, b, c) respectively containing 301, 367, and 541 particles respectively. 5 nm scale bars.

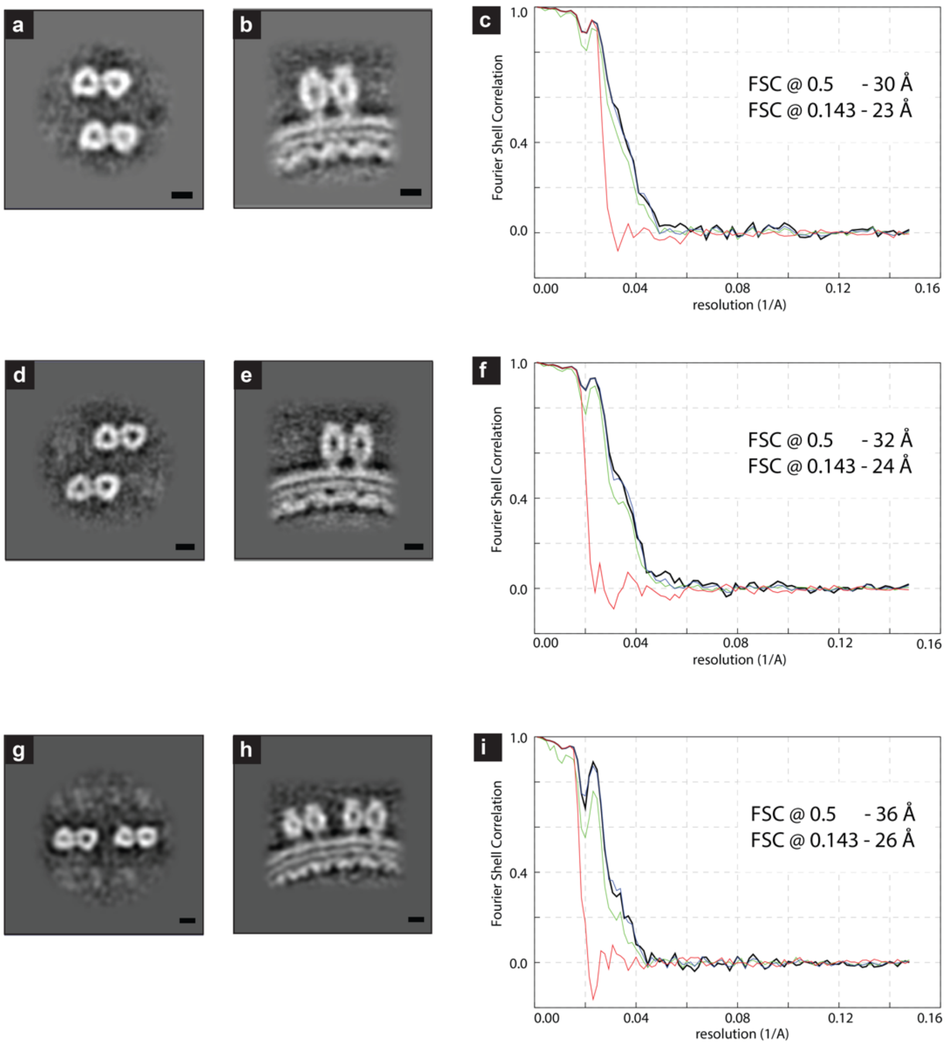

Supplementary Figure 9. FSC curves for sub-tomogram averages of higher-order F organizations. a, b Masked slices from sub-tomogram average containing the final average of four F-trimers. **c** Fourier shell correlation plot generated from Relion. Resolution is reported for FSC 0.5 and FSC 0.143 of the masked corrected plot (black line). **d, e, f** and **g, h, i** As for (a, b, c) for the two other averages containing different arrangements of F-trimers. 5 nm scale bar in (a), (b), (d), (e), (g), (h).

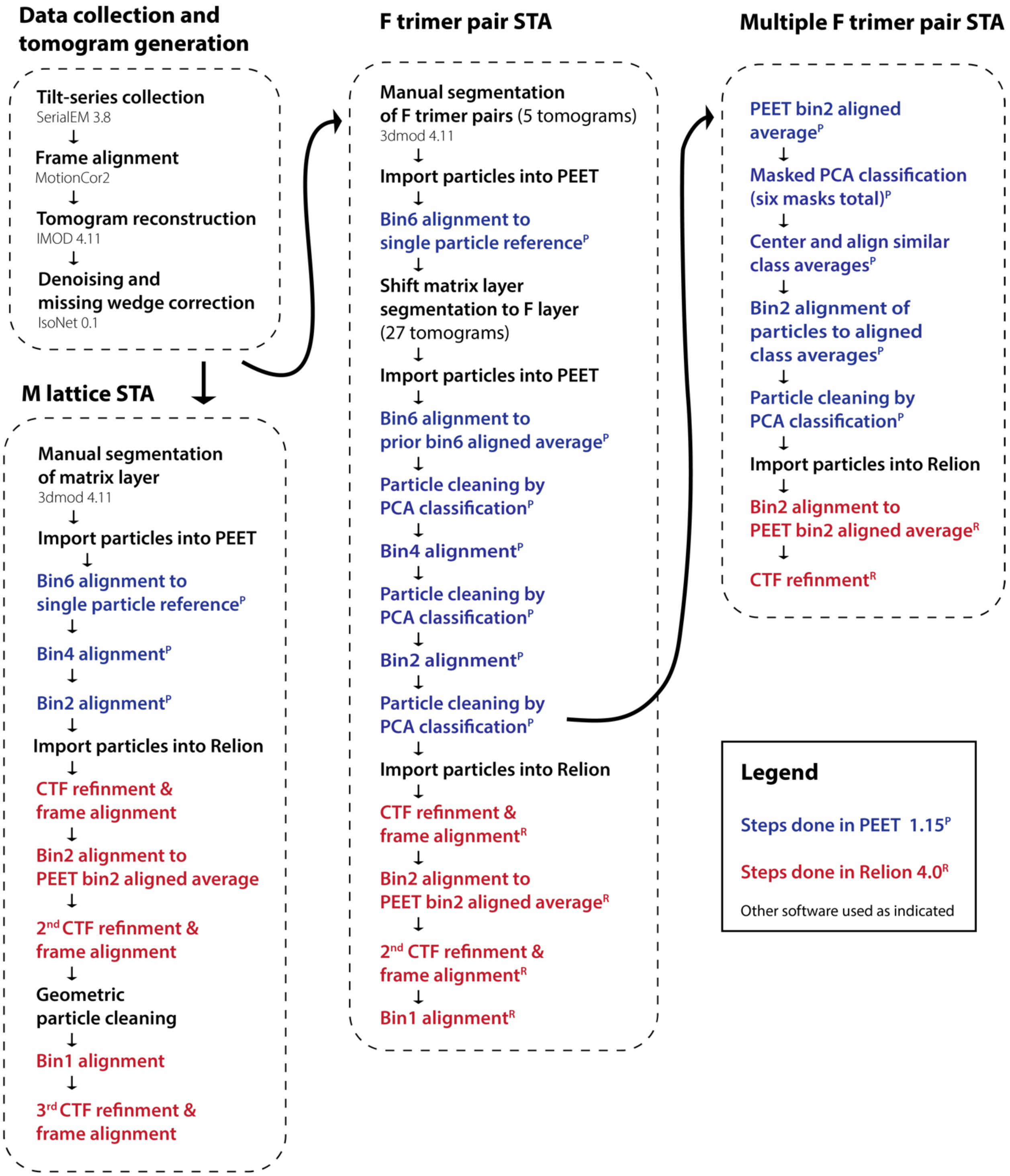
Data collection and tomogram generation
Tilt-series collection
SerialEM 3.8
Frame alignment
MotionCor2
Tomogram reconstruction
IMOD 4.11
Denoising and missing wedge correction
IsoNet 0.1
M lattice STA
Manual segmentation of matrix layer
3dmod 4.11
Import particles into PEET
Bin6 alignment to single particle reference^P
Bin4 alignment^P
Bin2 alignment^P
Import particles into Relion
CTF refinment & frame alignment
Bin2 alignment to PEET bin2 aligned average
2nd CTF refinment & frame alignment
Geometric particle cleaning
Bin1 alignment
3rd CTF refinment & frame alignment
F trimer pair STA
Manual segmentation of F trimer pairs (5 tomograms)
3dmod 4.11
Import particles into PEET
Bin6 alignment to single particle reference^P
Shift matrix layer segmentation to F layer (27 tomograms)
Import particles into PEET
Bin6 alignment to prior bin6 aligned average^P
Particle cleaning by PCA classification^P
Bin4 alignment^P
Particle cleaning by PCA classification^P
Bin2 alignment^P
Particle cleaning by PCA classification^P
Import particles into Relion
CTF refinment & frame alignment^R
Bin2 alignment to PEET bin2 aligned average^R
2nd CTF refinment & frame alignment^R
Bin1 alignment^R
Multiple F trimer pair STA
PEET bin2 aligned average^P
Masked PCA classification (six masks total)^P
Center and align similar class averages^P
Bin2 alignment of particles to aligned class averages^P
Particle cleaning by PCA classification^P
Import particles into Relion
Bin2 alignment to PEET bin2 aligned average^R
CTF refinment^R
Legend
Steps done in PEET 1.15^P
Steps done in Relion 4.0^R
Other software used as indicated

Supplementary Figure 10. Diagram of STA processing workflows. General steps of data processing and STA are shown in the flowchart above. Steps performed in PEET 1.5 are highlighted in blue with a superscript P and steps performed in Relion 4.0 are highlighted in red with superscript R. Other software packages used are indicated beneath the associated step.

Supplementary Table 1. Summary of matrix organization of selected viruses

Virus	Virion Morphology	Matrix Organization	Reference(s)	Structure(s)
**Respiratory Syncytial Virus**	Filamentous	Helical-like lattice	This work, 2024	EMD-44965
			Conley et al. 2022[20]	EMD-13855
**Ebola VLPs/Marburg Virus**	Filamentous	C2 symmetry	Wan et al., 2021[34]	EMD-11660, EMD-11661, EMD-11662, EMD-11663, EMD-11664
**Measles virus**	Oblate spherical	2D array with C4 symmetry	Ke and Strauss et al., 2018[63]	EMD-7565, EMD-7566
**Newcastle Disease Virus**	Oblate spherical	Grid-like array	Battisti et al., 2012[33]	EMD-5448
**Rabies Virus**	Bullet	Strings	Riedel et al., 2019[64]	EMD-4995
**Vesicular Stomatitis Virus**	Bullet	Two-layer helical array	Zhou et al., 2022[1]	EMD-26841
			Jenni et al., 2022[68]	EMD-26602, EMD-26603
**Influenza A Virus**	Spherical or Filamentous	Helical array	Peukes et al., 2020[35]	EMD-11077, EMD-11078, EMD-11079

Supplementary Table 2. Cryo-ET data collection parameters.

Microscope	TFS Titan Krios
Voltage (kV)	300
Energy Filter	Gatan Bioquantum, 20 eV slit
Detector	Gatan K3
Detector mode	Counting, non-CDS, non-super resolution
Frames per tilt	8
Magnification (x)	53,000
Pixel size (data collected) (Å)	1.693
Defocus (µm)	-2.0 to -6.0
Tilt series angle coverage (°)	-60 to + 60 (dose symmetric)
Tilt series increment angle (°)	3
Electron dose per tomogram (e^-/Å^2)	100

Supplementary Table 3. Sub-tomogram averaging of the matrix protein.

	Program	# of tomos	Initial reference	Input particle #	Retained particle #	Pixel size (Å)	Volume size (px)	Binary mask shape and dimensions (px)	Soft mask edge	Distance between particles (voxels)	Additional exclusion criteria
Round 1(a)	PEET	10	single particle from tomogram #2	110,296	36,561	10.158	48,48,48	Cylinder 21,10 (r,h)	Gaussian convolution StdDev 4 px	5	NA
Round 1(b)	PEET	10	single particle from tomogram #2	108,522	36,621	10.158	48,48,48	Cylinder 21,10 (r,h)	Gaussian convolution StdDev 4 px	5	NA
Round 1(c)	PEET	7	single particle from tomogram #2	87,171	29,363	10.158	48,48.48	Cylinder 21,10 (r,h)	Gaussian convolution StdDev 4 px	5	NA
Round 2(a)	PEET	14	Round 1(b) avg lowpass filtered 40 Å	51,897	37,127	6.772	64,64,64	Cylinder 28,14 (r,h)	Gaussian convolution StdDev 6 px	8	NA
Round 2(b)	PEET	13	Round 1(b) avg lowpass filtered 40 Å	50,648	37,371	6.772	64,64,64	Cylinder 28,14 (r,h)	Gaussian convolution StdDev 6 px	8	NA
Round 3(a)	PEET	14	Round 2(a) avg lowpass filtered 25 Å	37,127	34,817	3.386	96,96,96	Cylinder 42,28 (r,h)	Gaussian convolution StdDev 12 px	14	NA
Round 3(b)	PEET	13	Round 2(a) avg lowpass filtered 25 Å	37,371	33,058	3.386	96,96,96	Cylinder 42,28 (r,h)	Gaussian convolution StdDev 12 px	14	NA
Round 4	RELION	27	Average of particles from Round 3 a+b lowpass filterd 16 Å	67,875	38,771	3.386	96,96,96	Cylinder 33,18 (r,h)	Cosine shaped soft edge 8 px	NA	Geometric cleaning
Round 5	RELION	27	Average of particles from Round 4 lowpass filterd 6 Å	38,771	38,771	1.693	192,192, 192	Cylinder 62,35 (r,h)	Cosine shaped soft edge 8 px	NA	NA

Notes: Particles closer than minimum distance between particles were considered duplicates and removed with PEET removeDuplicates; r – radius (x, y), h – height (z).

Supplementary Table 4. Sub-tomogram averaging of F-pairs.

	Program	# of tomos	Initial reference	Input particle #	Retained particle #	Pixel size (Å)	Volume size (px)	Binary mask shape and dimensions (px)	Soft mask edge	Distance between particles (voxels)	Additional exclusion criteria
Round 1	PEET	5	single particle from tomogram #2	1031	1022	10.158	48,48,48	Cylinder 15,24 (r,h)	Gaussian convolution StdDev 4 px	4	NA
Round 2(a)	PEET	10	Round 1 avg lowpass filtered 50 Å	110,296	16,139	10.158	36,36,36	Cylinder 12,22 (r,h)	Gaussian convolution StdDev 6 px	5	PCA classification
Round 2(b)	PEET	10	Round 1 avg lowpass filtered 50 Å	108,522	19,537	10.158	36,36,36	Cylinder 12,22 (r,h)	Gaussian convolution StdDev 6 px	5	PCA classification
Round 2(c)	PEET	7	Round 1 avg lowpass filtered 50 Å	87,171	19,153	10.158	36,36,36	Cylinder 12,22 (r,h)	Gaussian convolution StdDev 6 px	5	PCA classification
Round 3(a)	PEET	14	Round 2(b) avg lowpass filtered 40 Å	25,860	12,227	6.772	54,54,54	Cylinder 18,33 (r,h)	Gaussian convolution StdDev 9 px	8	PCA classification
Round 3(b)	PEET	13	Round 2(b) avg lowpass filtered 40 Å	28,969	10,564	6.772	54,54,54	Cylinder 18,33 (r,h)	Gaussian convolution StdDev 9 px	8	PCA classification
Round 4(a)	PEET	14	Round 3(a) avg lowpass filtered 30 Å	12,227	11,428	3.386	96,96,96	Cylinder 36,66 (r,h)	Gaussian convolution StdDev 18 px	15	PCA classification
Round 4(b)	PEET	13	Round 3(a) avg lowpass filtered 30 Å	10,564	10,094	3.386	96,96,96	Cylinder 36,66 (r,h)	Gaussian convolution StdDev 18 px	15	PCA classification
Round 5	RELION	27	Average of particles from Round 4 a+b lowpass filterd 15 Å	21,522	21,522	3.386	128,128, 128	Cylinder 25,70 (r,h)	Cosine shaped soft edge 18 px	NA	NA
Round 6	RELION	27	Average of particles from Round 5 lowpass filterd 14 Å	21,522	21,522	1.693	192,192, 192	Cylinder 50,140 (r,h)	Cosine shaped soft edge 18 px	NA	NA

Notes: Particles closer than minimum distance between particles were considered duplicates and removed with PEET removeDuplicates; r – radius (x, y), h – height (z).

Supplementary Table 5. PCA Classification of multiple F-pairs.

	Program	Initial particles from	# of tomos	Input particle #	Retained particle #	Volume size (px)	Binary mask shape and dimensions (px)	Binary mask position (px)
PCA 1	PEET	F-pair Round 4(a)	14	11,428	301	96,128,96	Cuboid 62,34,60 (x,y,z)	10,83,18 (x,y,z)
PCA 2	PEET	F-pair Round 4(b)	13	10,094	415	96,128,96	Cuboid 62,34,60 (x,y,z)	10,83,18 (x,y,z)
PCA 3	PEET	F-pair Round 4(a)	14	11,428	410	96,128,96	Cuboid 62,34,60 (x,y,z)	24,5,49 (x,y,z)
PCA 4	PEET	F-pair Round 4(b)	13	10,094	351	96,128,96	Cuboid 62,34,60 (x,y,z)	24,5,49 (x,y,z)
PCA 5	PEET	F-pair Round 4(a)	14	11,428	367	144,128,96	Cuboid 62,34,60 (x,y,z)	60,86,18 (x,y,z)
PCA 6	PEET	F-pair Round 4(b)	13	10,094	385	144,128,96	Cuboid 62,34,60 (x,y,z)	60,86,18 (x,y,z)
PCA 7	PEET	F-pair Round 4(a)	14	11,428	892	144,128,96	Cuboid 62,34,60 (x,y,z)	20,4,18 (x,y,z)
PCA 8	PEET	F-pair Round 4(b)	13	10,094	460	144,128,96	Cuboid 62,34,60 (x,y,z)	20,4,18 (x,y,z)
PCA 9	PEET	F-pair Round 4(a)	14	11,428	541	196,96,96	Cuboid 62,34,70 (x,y,z)	126,31,8 (x,y,z)
PCA 10	PEET	F-pair Round 4(b)	13	10,094	371	196,96,96	Cuboid 62,34,70 (x,y,z)	126,31,8 (x,y,z)
PCA 11	PEET	F-pair Round 4(a)	14	11,428	422	196,96,96	Cuboid 62,34,70 (x,y,z)	9,25,1 (x,y,z)
PCA 12	PEET	F-pair Round 4(b)	13	10,094	714	196,96,96	Cuboid 62,34,70 (x,y,z)	9,25,1 (x,y,z)

Notes: Binary mask position defines the lower left corner of the mask in 3D with (1, 1, 1) as the lower left pixel in the volume.

Supplementary Table 6. Sub-tomogram averaging of multiple F-pairs.

	Program	# of tomos	Initial reference	Input particle #	Retained particle #	Pixel size (Å)	Volume size (px)	Binary mask shape and dimensions (px)	Soft mask edge	Distance between particles (voxels)	Additional exclusion criteria
Round 1(a)	PEET	Selected class averages from PCA 1-4	Class average from PCA 1	4	4	3.386	96,128, 96	Cylinder 42,66 (r,h)	Gaussian convolution StdDev 18 px	NA	NA
Round 1(b)	PEET	Selected class averages from PCA 5-8	Class average from PCA 5	4	4	3.386	144,128, 96	Cylinder 48,66 (r,h)	Gaussian convolution StdDev 18 px	NA	NA
Round 1(c)	PEET	Selected class averages from PCA 9-12	Class average from PCA 11	4	4	3.386	196,96, 96	Cylinder 58,66 (r,h)	Gaussian convolution StdDev 18 px	NA	NA
Round 2(a)	PEET	27	Round 1(a) avg lowpass filtered 50 Å	1,477	1,030	3.386	96,128, 96	Cylinder 42,66 (r,h)	Gaussian convolution StdDev 12 px	15	PCA classification
Round 2(b)	PEET	27	Round 1(b) avg lowpass filtered 50 Å	2,104	1,246	3.386	144,128, 96	Cylinder 48,66 (r,h)	Gaussian convolution StdDev 12 px	15	PCA classification
Round 2(c)	PEET	27	Round 1(c) avg lowpass filtered 50 Å	2,048	1,132	3.386	196,96, 96	Cylinder 58,66 (r,h)	Gaussian convolution StdDev 12 px	15	PCA classification
Round 3(a)	RELION	27	Average of particles from Round 2(a) lowpass filtered 60 Å	1,030	1,030	3.386	144,144, 144	Cylinder 38,80 (r,h)	Cosine shaped soft edge 18 px	NA	NA
Round 3(b)	RELION	27	Average of particles from Round 2(b) lowpass filtered 60 Å	1,246	1,246	3.386	160,160, 160	Cylinder 43,80 (r,h)	Cosine shaped soft edge 18 px	NA	NA
Round 3(c)	RELION	27	Average of particles from Round 2(c) lowpass filtered 60 Å	1,132	1,132	3.386	192,192, 192	Cylinder 56,80 (r,h)	Cosine shaped soft edge 18 px	NA	NA

Notes: Round 1 was an alignment of class averages following PCA classification to generate initial references for round 2 and did not include individual particles. Particles closer than minimum distance between particles were considered duplicates and removed with PEET removeDuplicates; r – radius (x, y), h – height (z).

SUPPLEMENTARY REFERENCES.

1. Zhou, K. et al. Atomic model of vesicular stomatitis virus and mechanism of assembly. *Nat. Commun.* **13**, 5980 (2022).
