# Supplementary material for: Assembly of respiratory syncytial virus matrix protein lattice and its coordination with fusion glycoprotein trimers
Source: Nat Commun. 2024 Jul 14;15:5923. doi: 10.1038/s41467-024-50162-x (PMC11247094; doi:10.1038/s41467-024-50162-x)
Supplement: Supplementary file 2 — Description of Additional Supplementary Files [file 41467_2024_50162_MOESM2_ESM.pdf]

Description of Additional Supplementary Files

File Name: Supplementary Movie 1
Description: Z-stack slice series, isosurface, and model fitting of M-lattice sub-tomogram average. XZ-slices of the M-lattice sub-tomogram average (Fig. 2) are shown as viewed with the membrane above matrix perpendicular to the viewing plane. The volume is rotated 90º around X and XY-slices of the density map are displayed moving in Z toward the virus interior. The isosurface is displayed and rotated as viewed from the outside of the virus. The isosurface is rotated 85º around X and 40º around Y to display the rows of U-shaped dimers comprising the lattice. The isosurface is then rotated to be displayed as viewed from the interior of the virus. Four ribbon diagrams of the M-dimer atomic model (PDB: 4v23) are shown docked into the sub-tomogram average. Individual monomers within each dimer are colored cyan and dark cyan. The isosurface is rotated back to be as viewed from outside the virus with membrane parallel to the imaging plane. 5 nm scale bar.

File Name: Supplementary Movie 2
Description: Z-stack slice series, isosurface, and model fitting of F pair sub-tomogram average. XZ-slices of the F-pair sub-tomogram average (Fig. 3) are shown as viewed with the F-pair above the membrane which is perpendicular to the viewing plane. The volume is rotated 90º around X and XY-slices of the density map are displayed moving in Z toward the virus interior. The isosurface is displayed as viewed from the outside of the virus. Ribbon diagrams of the F-trimer atomic model (PDB: 4JHW) are shown docked into the sub-tomogram average. The isosurface is rotated 90˚ around X and 90º around Y to display additional views of the sub-tomogram average and model-fitting. The isosurface is then rotated to be displayed as viewed from the interior of the virus. Six ribbon diagrams of the M-dimer atomic model (PDB: 4v23) are shown docked into the sub-tomogram average. The isosurface is rotated back to be as viewed from outside the virus with membrane parallel to the imaging plane. 5 nm scale bar.

File Name: Supplementary Movie 3
Description: Z-stack slice series and isosurface of a sub-tomogram average of four F trimers. XZ-slices of a four F trimer sub-tomogram average (Fig. 4a) are shown as viewed with the F-pair above the membrane which is perpendicular to the viewing plane. The volume is rotated 90º around X and XY-slices of the density map are displayed moving in Z toward the virus interior. The isosurface is displayed as viewed from the outside of the virus. The isosurface is rotated 90º around X and 90º around Y to display additional views of the sub-tomogram average. The isosurface is then rotated to be displayed as viewed from the interior of the virus. The isosurface is rotated back to be as viewed from outside the virus with membrane parallel to the imaging plane. 5 nm scale bar.

File Name: Supplementary Movie 4
Description: Z-stack slice series and isosurface of a sub-tomogram average of four F trimers. XZ-slices of a four F trimer sub-tomogram average (Fig. 4g) are shown as viewed with the F-pair above the membrane which is perpendicular to the viewing plane. The volume is rotated 90º around X and XY-slices of the density map are displayed moving in Z toward the virus interior. The isosurface is displayed as viewed from the outside of the virus. The isosurface is rotated 90º around X and 90º around Y to display additional views of the sub-tomogram average. The isosurface is then rotated to be displayed as viewed from the interior of the virus. The isosurface is rotated back to be as viewed from outside the virus with membrane parallel to the imaging plane. 5 nm scale bar.

File Name: Supplementary Movie 5

Description: Z-stack slice series and isosurface of a sub-tomogram average of four F trimers. XZ-slices of a four F trimer sub-tomogram average (Fig. 4m) are shown as viewed with the F-pair above the membrane which is perpendicular to the viewing plane. The volume is rotated 90º around X and XY-slices of the density map are displayed moving in Z toward the virus interior. The isosurface is displayed as viewed from the outside of the virus. The isosurface is rotated 90º around X and 90º around Y to display additional views of the sub-tomogram average. The isosurface is then rotated to be displayed as viewed from the interior of the virus. The isosurface is rotated back to be as viewed from outside the virus with membrane parallel to the imaging plane. 5 nm scale bar.
